# Association of a routine dexmedetomidine-remifentanil anesthetic strategy with POD3 morning cortisol change in older patients undergoing radical esophagectomy

**DOI:** 10.3389/fendo.2026.1911323

**Published:** 2026-09-25

**Authors:** Yuling Yang, Junbiao Chen, Zhicheng Lin, Lisha Yi, Nanye Zhu, Jiangquan Hong, Yimin Lin, Jiabin Li

**Affiliations:** 1 Department of Thoracic Surgery, Zhangzhou Affiliated Hospital of Fujian Medical University, Zhangzhou, Fujian, China; 2 Department of Clinical Laboratory, Zhangzhou Affiliated Hospital of Fujian Medical University, Zhangzhou, Fujian, China; 3 Department of Gastroenterology, Zhangzhou Affiliated Hospital of Fujian Medical University, Zhangzhou, Fujian, China; 4 Department of Anesthesiology, Zhangzhou Affiliated Hospital of Fujian Medical University, Zhangzhou, Fujian, China

**Keywords:** ACTH, cortisol, dexmedetomidine, esophageal cancer, esophagectomy, HPA axis, postoperative neurocognitive decline, remifentanil

## Abstract

**Background:**

Older patients undergoing radical esophagectomy may experience marked postoperative endocrine stress, inflammation, pain, and short-term cognitive decline. We evaluated whether a routine anesthetic strategy combining dexmedetomidine with remifentanil was associated with a smaller POD3 serum cortisol increase than remifentanil- or dexmedetomidine-based strategies alone.

**Methods:**

This single-center retrospective cohort screened 426 consecutive patients aged ≥65 years who underwent elective radical esophagectomy from January 2023 to December 2025. Of these, 347 had verifiable pathway-based POD3 biomarker and POD7 MoCA data after predefined clinical, laboratory, and follow-up exclusions. The primary analysis used a prespecified balanced common-support analytic cohort drawn from the naturally occurring REM (continuous remifentanil without dexmedetomidine), DEX (dexmedetomidine with non-remifentanil opioid analgesia and without continuous remifentanil), and Dex-REM (both infusions) exposure strata (100 patients each). The primary endpoint was morning serum cortisol change from preoperative day 1 to postoperative day 3 (POD3).

**Results:**

POD7 screening-based cognitive decline occurred in 51 patients in the balanced common-support cohort. Mean cortisol change was +164.0 nmol/L in REM, + 126.0 nmol/L in DEX, and +86.0 nmol/L in Dex-REM. After adjustment, Dex-REM was associated with lower cortisol change than REM (adjusted mean difference, -78.4 nmol/L; 95% CI, -103.2 to -53.6; p<0.001) and DEX (-39.6 nmol/L; 95% CI, -64.1 to -15.1; p=0.002). Dex-REM also showed lower 24-h VAS AUC than REM (adjusted mean difference, -5.8 point-hours; 95% CI, -9.3 to -2.3; FDR q=0.006), while POD7 MoCA-decline risk was numerically lower but imprecise (12.0% vs. 22.0%; adjusted risk ratio, 0.52; 95% CI, 0.26 to 1.05; p=0.071).

**Conclusions:**

The real-world Dex-REM anesthetic pathway was associated with a smaller POD3 morning serum cortisol increase. This pathway-level association does not establish an agent-specific effect, pharmacologic synergy, or attenuation of the complete surgical-stress trajectory. Clinical and screening-based cognitive secondary outcomes were exploratory and require prospective multicenter confirmation before causal or neuroprotective conclusions are drawn.

## Introduction

Esophageal cancer remains a major global cancer burden, and contemporary GLOBOCAN estimates emphasize its continuing contribution to cancer mortality worldwide (1). Curative-intent radical esophagectomy remains central for appropriately selected patients, often within multimodality strategies guided by current esophageal cancer recommendations (2, 3). As populations age, a growing proportion of surgical candidates are older adults with comorbidity, reduced physiologic reserve, and heightened vulnerability to postoperative complications; contemporary elderly esophagectomy cohorts and systematic-review evidence support surgery in selected patients but emphasize the need for careful perioperative optimization (4, 5). Enhanced-recovery guidance for esophagectomy also highlights the importance of standardized perioperative pathways (6). For older patients, the clinical objective is therefore not only technically successful resection, but also attenuation of avoidable endocrine, inflammatory, analgesic, and cognitive stressors during the immediate recovery window.

Major thoracoabdominal surgery triggers an integrated stress response involving sympathetic activation, Hypothalamic-pituitary-adrenal (HPA) axis signaling, inflammatory cytokine release, metabolic mobilization, and immune modulation (7, 8). Anesthesia and analgesia can shape this response through depth of anesthesia, opioid exposure, nociceptive control, autonomic tone, and hemodynamic stability (9). Cortisol is a clinically accessible HPA-axis marker, and surgical cortisol responses vary by surgical severity, anesthetic technique, patient characteristics, sampling time, and postoperative complications (10). Cortisol is routinely available and has an established perioperative evidence base for characterizing surgical stress responses (10). Radical esophagectomy is particularly relevant to an endocrine-stress-centered analysis because it combines prolonged anesthesia, tissue trauma, one-lung ventilation in many cases, postoperative pain, and high inflammatory burden. The present study therefore used POD3 morning cortisol change as a pragmatic postoperative biomarker.

Dexmedetomidine is a highly selective alpha-2 adrenergic agonist with sedative, sympatholytic, anxiolytic, opioid-sparing, and modest analgesic properties (11). Meta-analysis suggests that perioperative dexmedetomidine can attenuate stress and inflammatory responses in surgical patients (12). Observational and randomized evidence also links perioperative dexmedetomidine with lower postoperative delirium or neurocognitive risk in older non-cardiac surgical populations, although findings remain context-dependent and safety concerns such as bradycardia and hypotension require close attention (13, 14). Remifentanil offers potent short-acting intraoperative opioid analgesia with rapid titratability, but intense or prolonged exposure has been associated with postoperative hyperalgesia or greater acute pain and rescue analgesic requirements in some settings (15, 16), and comparative analgesia evidence suggests that perioperative effects depend strongly on dose and context (17). These pharmacologic profiles provide a biologically plausible rationale for a combined dexmedetomidine-remifentanil strategy; however, in observational data this strategy should be interpreted as a clinical anesthetic pathway with different opioid mixtures and doses, not as proof of pharmacologic synergy.

The neurocognitive dimension further strengthens the rationale for an endocrine-stress-centered perioperative study. Current consensus recommends using perioperative neurocognitive-disorder terminology rather than older imprecise labels when cognitive change is assessed around anesthesia and surgery (18). Recent reviews describe postoperative neurocognitive disorders in older adults as multifactorial outcomes involving baseline cognitive reserve, inflammation, pain, sleep disruption, anesthetic exposure, and surgical burden (19). Inflammation may be especially relevant: delayed resolution of postoperative interleukin-6 has been associated with delayed cognitive recovery after surgery (20). MoCA is a brief cognitive screening tool, and a 2-point postoperative decrease has been used operationally in perioperative studies, but this threshold is not equivalent to a comprehensive neuropsychological diagnosis (21, 22). A design that jointly evaluates cortisol, ACTH, inflammatory response, pain burden, hemodynamic variability, and screening-based early cognitive change may therefore provide a coherent perioperative recovery model while still requiring cautious interpretation.

The present retrospective single-center cohort study was designed to address this gap in older patients undergoing radical esophagectomy. We compared routine remifentanil-based anesthesia, dexmedetomidine-based anesthesia with non-remifentanil opioid analgesia, and combined dexmedetomidine-remifentanil anesthesia, with change in morning serum cortisol from preoperative day 1 to POD3 prespecified as the single primary endpoint. We hypothesized that the combined Dex-REM strategy would be associated with a smaller POD3 cortisol increase than REM alone, and that ACTH, inflammatory, pain, hemodynamic, and screening-based early cognitive outcomes would provide supportive, exploratory recovery information. By assigning one endocrine primary outcome, treating binary neurocognitive outcomes as secondary, and using parsimonious adjustment, the study was structured to avoid treating a moderate complete-case retrospective cohort as definitive causal evidence.

## Methods

### Study design and setting

This retrospective observational cohort study was conducted at the Zhangzhou Affiliated Hospital of Fujian Medical University and followed the STROBE and RECORD reporting frameworks for observational studies using routinely collected health data (23, 24). Consecutive patients aged 65 years or older undergoing elective radical esophagectomy for esophageal cancer between January 2023 and December 2025 were first identified from the thoracic surgery schedule, anesthesia information management system, electronic medical record, laboratory information system, post-anesthesia care unit records, and standardized postoperative follow-up forms. During the study period, older esophagectomy patients were managed through an institutional high-risk thoracic-surgery perioperative pathway that prospectively recorded preoperative and POD7 MoCA assessments, early-morning POD3 endocrine/inflammatory panels, anesthetic infusion records, and recovery forms as routine quality data. The study was non-interventional: anesthetic regimens were selected by the responsible anesthesia team according to routine institutional practice, and the analysis compared naturally occurring exposure groups documented before anesthetic induction. The primary analysis used a prespecified 1:1:1 balanced common-support analytic cohort selected from eligible complete-data cases; the full eligible cohort was retained for overlap-weighted sensitivity analysis.

This study was conducted in accordance with the Declaration of Helsinki. The protocol was reviewed and approved by the Ethics Committee of the Zhangzhou Affiliated Hospital of Fujian Medical University, which waived the requirement for informed consent because this was a retrospective analysis of de-identified routinely collected data and posed no more than minimal risk to participants. All data were anonymized prior to analysis.

### Participants and time zero

Eligible patients were aged 65 years or older, had pathologically or clinically diagnosed esophageal cancer, underwent elective radical esophagectomy under general anesthesia, had ASA physical status II or III, had serum cortisol measurement within 24 h before surgery and on POD3, and had documented baseline and POD7 cognitive assessment from the institutional perioperative pathway. Exclusion criteria were emergency surgery, palliative operation only, combined major surgery unrelated to esophagectomy, preoperative dementia, Parkinson disease with cognitive impairment, active psychosis, recent stroke, inability to complete cognitive testing, chronic systemic glucocorticoid therapy, known pituitary or adrenal disease, Cushing syndrome, adrenal insufficiency, severe thyroid dysfunction likely to distort HPA-axis biomarkers, severe hepatic failure, end-stage renal disease, uncontrolled preoperative infection, immunosuppressive therapy other than standard oncologic treatment, missing exposure records, or conversion to a non-study anesthetic plan before induction. After eligibility screening, patients entered the natural exposure stratum documented before induction; a balanced 1:1:1 common-support analytic cohort was then created by selecting 100 patients from each stratum. Patients not selected during common-support selection were not treated as clinical exclusions and contributed to the full-cohort overlap-weighted sensitivity analysis. Time zero was the start of anesthetic induction in the operating room, and exposure group assignment had to be documented by or before this time, consistent with target-trial principles for avoiding immortal time bias and reverse causation (25). Requiring POD3 cortisol and POD7 cognitive data created a complete-case pathway cohort; although the screening log now reports major missingness and clinical-instability pathways, this design may still have underrepresented patients who were too ill, transferred, discharged, or otherwise unable to complete follow-up testing.

### Exposure definitions and perioperative data

The primary exposure was the routine anesthetic strategy documented in the pre-induction anesthesia plan and verified from infusion records. The REM group received continuous remifentanil infusion as the principal short-acting intraoperative opioid component without dexmedetomidine infusion; the DEX group received dexmedetomidine infusion as a sedative or sympatholytic adjunct without continuous remifentanil infusion, with fentanyl, sufentanil, or morphine-based intraoperative analgesia recorded separately; and the Dex-REM group received both dexmedetomidine and remifentanil infusion during induction or maintenance according to institutional practice. The grouping variable was defined by the presence or absence of continuous intraoperative remifentanil and dexmedetomidine in the pre-induction plan and verified infusion record. The DEX category therefore denotes dexmedetomidine with non-remifentanil opioid analgesia, not dexmedetomidine monotherapy or opioid-free anesthesia. Conversion to sufentanil-equivalent dose permitted a common quantitative summary but did not make the opioid agents pharmacologically interchangeable. These groups were real-world anesthetic strategies, and no nonlinear dose-response or synergy model was prespecified. The estimand was the association of routine anesthetic pathways with outcomes, not the isolated effect of either agent or a pharmacologic synergy effect. All groups otherwise received institutional general anesthesia and routine postoperative analgesia according to clinical need; non-remifentanil opioids were converted to sufentanil-equivalent intraoperative dose, and postoperative rescue opioids were converted to morphine equivalents. This was routine care, induction agent, volatile versus intravenous maintenance, MAC/BIS target, neuromuscular-blocking and reversal agents, intravenous lidocaine, and magnesium were not available as complete standardized study variables. Any recorded systemic corticosteroid exposure before the POD3 blood draw was identifiable from medication records, but agent, dose, and indication were not complete standardized fields and were addressed through an exclusion sensitivity analysis. Regional analgesia was available only as a binary use/no-use variable; technique, local anesthetic, concentration, dose, timing, single-shot versus catheter status, and postoperative catheter continuation were not sufficiently complete for technique-specific analysis. Postoperative analgesia followed routine clinical care and was not uniform for this observational study. The consistently analyzable fields were NSAID/acetaminophen administration within 24 h and 24-h rescue opioid dose converted to morphine equivalents; complete PCA, epidural-infusion, rescue-algorithm, and drug-by-drug exposure trajectories through POD3 were unavailable. Exposure classification was restricted to intraoperative infusion records; postoperative continuation of dexmedetomidine was not available as a validated study variable. Dose variables included cumulative dexmedetomidine dose in micrograms/kg, mean dexmedetomidine infusion rate in micrograms/kg/h, active dexmedetomidine infusion duration, cumulative remifentanil dose in micrograms/kg, mean remifentanil infusion rate in micrograms/kg/min, and active remifentanil infusion duration. Mean infusion rates were calculated over actual active infusion time, not total anesthesia duration, so cumulative dose, infusion rate, and active infusion duration were interpreted together.

### Outcomes and measurement timing

The primary endpoint was change in morning serum cortisol from preoperative day 1 to POD3, expressed in nmol/L. Morning serum cortisol change was prespecified as the primary endpoint because cortisol is routinely available and has an established perioperative evidence base (10); ACTH was designated a mechanistically informative secondary marker. To reduce diurnal measurement bias, endocrine blood sampling was scheduled between 06:00 and 08:00 whenever clinically feasible, off-window samples were recorded when present, and the same laboratory platform was used throughout the study period. POD3 was selected *a priori* from the standardized early-morning endocrine panel embedded in the institutional pathway was obtained on that day and was intended to characterize endocrine status during early postoperative recovery after the immediate intraoperative/PACU period. It was not intended to estimate the perioperative peak or the complete HPA-axis trajectory. The institutional pathway specified cortisol, ACTH, catecholamines, CRP, IL-6, and routine inflammatory markers at baseline and POD3 for older radical esophagectomy patients; values used in the study required verifiable laboratory timestamps and assay records. The primary contrast was Dex-REM versus REM; the hierarchical secondary contrast Dex-REM versus DEX was interpreted only if the primary contrast was statistically significant. Key endocrine secondary outcomes were change in plasma ACTH from preoperative day 1 to POD3, catecholamine changes, and an exploratory HPA-axis stress burden score. The HPA-axis stress burden score was calculated as [z(delta cortisol) + z(delta ACTH)]/2, where each z score used the mean and SD of the balanced common-support cohort change-score distribution; the score is unitless, with higher values indicating greater combined HPA-axis activation, and was not treated as a validated diagnostic biomarker. Clinical secondary outcomes included CRP and IL-6 changes, 24-h VAS pain area under the curve calculated by trapezoidal integration of pathway pain scores during the first 24 h, PACU agitation, wake-up time defined as minutes from anesthetic discontinuation to extubation or reliable command response as recorded in the anesthesia record, hemodynamic time-weighted absolute MAP deviation from the pre-induction baseline, screening-based early postoperative cognitive decline, and in-hospital safety events. MoCA was used for cognitive screening (21), pain intensity was assessed using VAS (26), and POD7 early cognitive decline was defined as a decrease of at least 2 MoCA points from baseline on POD7 or before discharge if discharge occurred earlier (22). This POD7 endpoint was interpreted as a screening-based cognitive decline outcome, not as a formal perioperative neurocognitive-disorder diagnosis. Formal serial postoperative sedation scores were not part of the pathway; wake-up time and PACU agitation were available but were not treated as substitutes for a validated sedation scale. Safety outcomes were defined before analysis as follows: hypotension was MAP <65 mmHg or vasopressor treatment for hypotension; bradycardia was heart rate <50 beats/min or atropine treatment; respiratory depression was respiratory rate <8 breaths/min, SpO_2_ <90% despite oxygen therapy, or naloxone use; delayed emergence was failure to extubate or respond reliably within 30 min after anesthetic discontinuation; postoperative nausea/vomiting required antiemetic treatment; ICU transfer was unplanned postoperative ICU admission or escalation; and serious adverse events included death, reoperation, life-threatening deterioration, or unplanned ICU escalation during hospitalization.

### Covariates, missing data, and bias control

Prespecified covariates were selected because they plausibly influenced both anesthetic regimen choice and postoperative endocrine or cognitive outcomes. Core covariates included age, sex, body mass index, education years for cognitive analyses, ASA class, smoking status, hypertension, diabetes, coronary artery disease, chronic obstructive pulmonary disease, baseline hemoglobin, albumin, baseline cortisol, baseline ACTH, baseline MoCA score, tumor stage, neoadjuvant therapy, planned surgical approach, and calendar period. Multinomial propensity scores were estimated using pre-exposure and planning variables including age, ASA class, hypertension, baseline hemoglobin, baseline cortisol, baseline MoCA, tumor stage, neoadjuvant therapy, planned surgical approach, and calendar period. Patients outside the overlapping range of the three propensity-score distributions were excluded, and the balanced common-support cohort was selected by minimizing the maximum pairwise absolute standardized mean difference across the listed variables until 100 patients per stratum were retained. This procedure created a balanced common-support cohort rather than matched triplets; therefore, regression models were not stratified by matched set. Post-selection balance is reported using maximum pairwise absolute standardized mean differences. Variables with residual ASMD >0.20 after common-support selection, namely ASA class, hypertension, clinical tumor stage, and baseline hemoglobin, were forced into the adjusted outcome models. The primary endocrine model adjusted for baseline cortisol, age, sex, ASA class, hypertension, clinical tumor stage, baseline hemoglobin, neoadjuvant therapy, and planned surgical approach; operation duration, blood loss, transfusion, vasopressor use, and regional analgesia were evaluated in sensitivity models because they occur after time zero or reflect intraoperative course. Postoperative complications, postoperative inflammation, and rescue opioid use were not included in the primary endocrine model because they may lie on the causal pathway or act as colliders. Because endpoint availability was an inclusion criterion, POD3 cortisol and POD7 cognitive outcomes were not imputed; this complete-case design was treated as a limitation. Covariate missingness below 5% was handled by complete-case analysis, while covariate missingness of 5% or higher would be handled with multiple imputation using chained equations with at least 20 imputed datasets (27, 28).

### Sample size and power rationale

The primary sample size was fixed at 300 balanced common-support analytic cases, with 100 per exposure group, selected from 347 eligible complete-data cases. The equal group sizes therefore reflected prespecified balanced common-support cohort construction. With 100 patients per group and a two-sided alpha of 0.05, the study had approximately 80% power to detect a standardized mean difference of 0.40 and approximately 89% power to detect a standardized mean difference of 0.45 in the continuous primary cortisol-change endpoint. Baseline-adjusted analysis was expected to improve precision if baseline and POD3 cortisol were correlated. By contrast, POD7 early cognitive decline occurred in 51 patients, supporting parsimonious secondary models that included exposure group and a limited set of core covariates.

### Statistical analysis

Continuous variables were summarized as mean (standard deviation, SD) or median [interquartile range, IQR] according to distribution, and categorical variables were summarized as n (%). Baseline comparisons used analysis of variance, Kruskal-Wallis tests, or chi-square tests as appropriate, with p-values interpreted descriptively and absolute standardized mean differences used as the primary balance diagnostic; residual ASMD >0.20 was explicitly reported and handled in adjusted models. The primary analysis used linear regression for cortisol change with exposure group as the main predictor, mandatory adjustment for baseline cortisol, and the prespecified covariate set including the residual imbalance variables. Adjusted mean differences, 95% confidence intervals (CIs), and p-values were reported. ACTH change and the HPA-axis stress burden score were analyzed using the same model family. Robust HC3 standard errors were used as a sensitivity analysis, and no nonlinear dose-response exposure model was used. Repeated VAS and MAP measurements were examined using linear mixed-effects models with group, time, group-by-time interaction, and random patient intercepts, while VAS AUC and MAP time-weighted deviation provided the main interpretable summary measures. POD7 early cognitive decline was analyzed using modified Poisson regression with robust variance (29), with Firth logistic regression prespecified if sparse cells or convergence problems occurred (30, 31). Sensitivity analyses included a model additionally adjusted for operation duration, blood loss, transfusion, vasopressor use, and regional analgesia; exclusion of perioperative systemic steroid exposure; exclusion of major complications before POD3; robust standard errors; and modeling POD3 cortisol with baseline adjustment. Multinomial propensity-score overlap weighting was performed in the full eligible cohort using generalized propensity scores for the three treatment groups, with covariate balance summarized using maximum absolute standardized mean difference (32, 33). The single primary endpoint used hierarchical testing. Secondary endocrine outcomes included ACTH, HPA-axis stress burden, epinephrine, and norepinephrine; secondary clinical outcomes included VAS AUC, CRP, IL-6, and MAP time-weighted deviation. These secondary families used Benjamini-Hochberg false-discovery-rate control (34), while the POD7 cognitive screening endpoint was reported separately as an exploratory binary outcome. Linear-model residuals, influential observations, robust standard errors, variance inflation factors, and propensity-weight balance were checked before final reporting. Analyses were performed using R version 4.4 (35).

## Results

### Cohort construction and baseline profile

Among 426 consecutive source patients aged ≥65 years who underwent elective radical esophagectomy during the study period, 79 did not enter the eligible complete-data pathway cohort: missing or off-window POD3 cortisol (n=24), missing or invalid POD7 MoCA assessment (n=22), postoperative clinical instability, prolonged ventilation, unplanned ICU escalation, or death before planned POD7 assessment (n=18), discharge or transfer before valid cognitive follow-up (n=9), and incomplete pre-induction exposure documentation or non-study anesthetic conversion (n=6). Reliable exposure-group assignments for all of these 79 patients were not available. Among the remaining 347 eligible patients with verifiable biomarker and POD7 cognitive data, the natural exposure distribution before common-support selection was REM n=121, DEX n=106, and Dex-REM n=120. The prespecified balanced common-support analytic cohort included 300 patients, with 100 patients each in REM, DEX, and Dex-REM; 47 eligible complete-data patients were not selected during common-support selection and were retained only for sensitivity analysis (**Figure 1**). Baseline characteristics were similar for many variables, including age, sex, baseline MoCA, baseline cortisol, and baseline ACTH (**Table 1**). Baseline albumin was also similar across strategies (REM, 38.5 ± 4.1 g/L; DEX, 39.1 ± 4.4 g/L; Dex-REM, 38.7 ± 4.0 g/L; descriptive p=0.583; maximum pairwise ASMD = 0.14). However, residual post-selection imbalance remained for ASA physical status III (ASMD 0.27), hypertension (ASMD 0.30), clinical tumor stage (ASMD 0.30), and hemoglobin (ASMD 0.26); these variables were explicitly included in adjusted outcome models.

**Figure 1:**
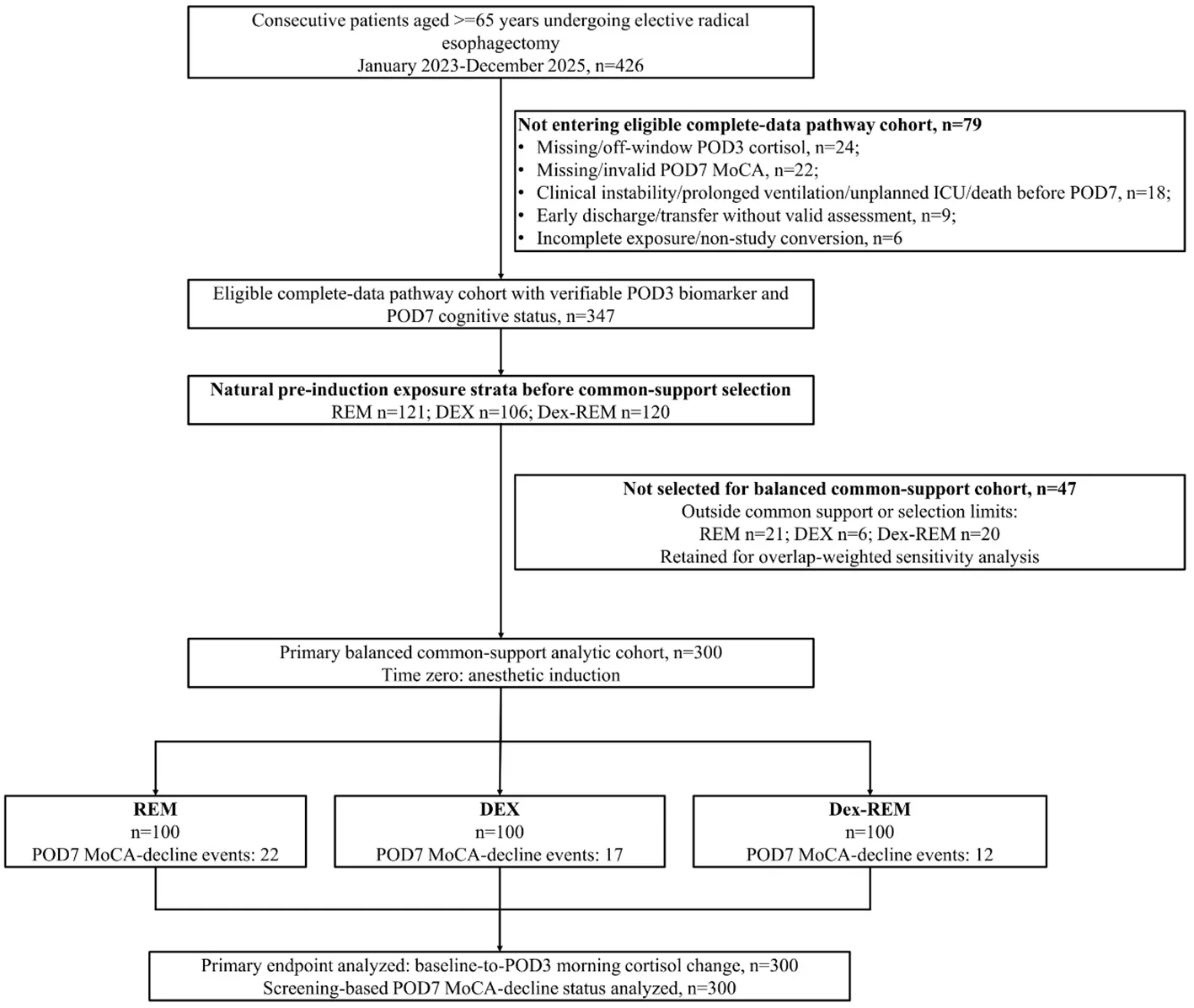
Study flowchart for the study cohort. The flow diagram starts from all consecutive older patients undergoing elective radical esophagectomy during the study period, shows pre-eligibility exclusions and missing follow-up pathways, natural exposure strata before common-support selection, patients not selected into the balanced analytic cohort, final exposure groups, the primary cortisol endpoint, and screening-based POD7 MoCA-decline events. Time zero was the start of anesthetic induction.

**Table 1:** Baseline characteristics in the balanced common-support cohort.

Characteristic	REM (n=100)	DEX (n=100)	Dex-REM (n=100)	p-value	Max pairwise ASMD
Age, years	71.7 (3.8)	71.3 (2.9)	71.6 (3.3)	0.664	0.12
Male sex	69 (69.0)	66 (66.0)	66 (66.0)	0.873	0.06
Body mass index, kg/m^2^	22.3 (2.9)	22.7 (2.8)	22.2 (2.7)	0.310	0.18
Education, years	9.6 (3.2)	9.7 (2.9)	9.3 (2.8)	0.654	0.14
ASA physical status III	48 (48.0)	35 (35.0)	36 (36.0)	0.112	0.27
Hypertension	50 (50.0)	40 (40.0)	55 (55.0)	0.097	0.30
Diabetes mellitus	26 (26.0)	21 (21.0)	18 (18.0)	0.382	0.19
Coronary artery disease	18 (18.0)	14 (14.0)	11 (11.0)	0.366	0.20
COPD	16 (16.0)	20 (20.0)	21 (21.0)	0.635	0.13
Current smoker	45 (45.0)	42 (42.0)	43 (43.0)	0.909	0.06
Neoadjuvant therapy	41 (41.0)	33 (33.0)	39 (39.0)	0.478	0.17
Clinical tumor stage I/II/III	15/42/43	11/42/47	22/38/40	0.319	0.30
Surgical approach open/hybrid/minimally invasive	22/28/50	20/29/51	19/27/54	0.976	0.08
Baseline MoCA score	25.1 (2.0)	24.8 (2.2)	25.0 (2.1)	0.581	0.14
Baseline cortisol, nmol/L	437.2 (61.4)	431.6 (58.8)	439.1 (64.2)	0.675	0.12
Baseline ACTH, pg/mL	28.9 (5.9)	28.1 (6.1)	28.6 (6.5)	0.635	0.13
Hemoglobin, g/L	124.1 (12.6)	123.1 (13.4)	126.6 (13.3)	0.155	0.26
Albumin, g/L	38.5 (4.1)	39.1 (4.4)	38.7 (4.0)	0.583	0.14

Values are mean (SD), median [IQR], or n (%). P-values are descriptive because treatment assignment was observational. The ASMD column reports the maximum pairwise absolute standardized mean difference across the three groups using displayed summaries; residual imbalances with ASMD >0.20 were ASA physical status III, hypertension, clinical tumor stage, and hemoglobin, and these variables were forced into adjusted outcome models. Equal group sizes reflect the prespecified balanced common-support analytic cohort, not naturally equal retrospective exposure frequencies.

### Exposure separation and perioperative course

Dexmedetomidine exposure was absent in REM and present in DEX and Dex-REM, with cumulative doses of 0.00, 1.68, and 1.52 micrograms/kg. Remifentanil exposure was present in REM and Dex-REM but absent as continuous infusion in DEX, with cumulative doses of 46.3, 0.0, and 37.8 micrograms/kg; infusion rates were calculated over active infusion time and were therefore consistent with the reported active infusion durations. These exposure-defining medication rows were not interpreted through significance tests. The DEX group received more non-remifentanil intraoperative opioid than REM or Dex-REM (0.41 vs. 0.19 and 0.24 micrograms/kg sufentanil-equivalent; p<0.001), confirming that DEX was not an opioid-free comparator and that the groups represented heterogeneous anesthetic pathways. Any regional analgesia was recorded in 45.0%, 56.0%, and 54.0% of REM, DEX, and Dex-REM patients (descriptive p=0.253), while NSAID/acetaminophen within 24 h was recorded in 89.0%, 91.0%, and 90.0% (p=0.895); technique-specific regional distributions could not be assessed (**Table 2**). The corresponding 24-h rescue-opioid doses were 10.1, 8.7, and 7.5 mg morphine equivalents (p<0.001). Operation duration, anesthesia duration, blood loss, transfusion, and vasopressor use were also similar across groups.

**Table 2:** Anesthetic strategies, co-interventions, and perioperative descriptors.

Characteristic	REM (n=100)	DEX (n=100)	Dex-REM (n=100)	p-value/note
Dexmedetomidine cumulative dose, micrograms/kg	0.00 (0.00)	1.68 (0.38)	1.52 (0.36)	Exposure-defining; not tested
Dexmedetomidine active infusion duration, min	0 (0)	229 (44)	228 (42)	Exposure-defining; not tested
Dexmedetomidine infusion rate, micrograms/kg/h	0.00 (0.00)	0.43 (0.10)	0.40 (0.09)	Exposure-defining; not tested
Remifentanil cumulative dose, micrograms/kg	46.3 (12.2)	0.0 (0.0)	37.8 (11.0)	Exposure-defining; not tested
Remifentanil active infusion duration, min	333 (49)	0 (0)	332 (46)	Exposure-defining; not tested
Remifentanil infusion rate, micrograms/kg/min	0.134 (0.035)	0.000 (0.000)	0.111 (0.032)	Exposure-defining; not tested
Non-remifentanil intraoperative opioid, sufentanil-equivalent micrograms/kg	0.19 (0.07)	0.41 (0.11)	0.24 (0.08)	<0.001
Any regional analgesia used	45 (45.0)	56 (56.0)	54 (54.0)	0.253
NSAID or acetaminophen within 24 h	89 (89.0)	91 (91.0)	90 (90.0)	0.895
Operation duration, min	309 (48)	305 (48)	305 (44)	0.831
Anesthesia duration, min	351 (51)	345 (49)	349 (45)	0.680
Estimated blood loss, mL	298 [204, 368]	312 [231, 374]	328 [238, 408]	0.403
Intraoperative transfusion	17 (17.0)	11 (11.0)	9 (9.0)	0.201
Intraoperative vasopressor use	30 (30.0)	34 (34.0)	29 (29.0)	0.721
24-h rescue opioid, morphine equivalents mg	10.1 (4.8)	8.7 (4.0)	7.5 (4.6)	<0.001
Wake-up time, min	19.1 (5.3)	17.2 (5.0)	15.2 (5.0)	<0.001
PACU agitation event	15 (15.0)	10 (10.0)	6 (6.0)	0.111

Values are mean (SD), median [IQR], or n (%). Infusion rates are calculated over actual active infusion time, not total anesthesia duration. Drug-exposure rows with structural zeros define the exposure strategies and were not interpreted through significance testing. Non-remifentanil intraoperative opioids were converted to sufentanil-equivalent dose; postoperative rescue opioids were converted to morphine equivalents. Rescue opioid use, wake-up time, and PACU agitation are post-exposure recovery descriptors. DEX denotes dexmedetomidine without continuous remifentanil and includes non-remifentanil opioid analgesia; it is not an opioid-free group. Regional analgesia was recorded only as use versus no use, and technique-level data were unavailable.

### Primary and key endocrine outcomes

With exposure separation documented, the primary analysis showed a smaller POD3 cortisol increase in Dex-REM. Cortisol increased from 437.2 to 601.2 nmol/L in REM, from 431.6 to 557.6 nmol/L in DEX, and from 439.1 to 525.1 nmol/L in Dex-REM, corresponding to mean changes of +164.0, +126.0, and +86.0 nmol/L (**Figure 2**). In the adjusted primary model, Dex-REM was associated with lower cortisol change than REM (aMD, -78.4 nmol/L; 95% CI, -103.2 to -53.6; p<0.001), and the hierarchical Dex-REM versus DEX contrast was also significant (aMD, -39.6 nmol/L; 95% CI, -64.1 to -15.1; p=0.002). ACTH change showed the same direction, with an adjusted Dex-REM versus REM difference of -8.5 pg/mL (95% CI, -10.8 to -6.2; FDR q<0.001). The exploratory HPA-axis stress burden score was lower (beta, -0.92 SD; 95% CI, -1.17 to -0.67; FDR q<0.001), but this unitless composite was interpreted only as supportive biological consistency. Catecholamine changes were weaker and were not treated as independent confirmatory evidence (**Table 3**).

**Figure 2:**
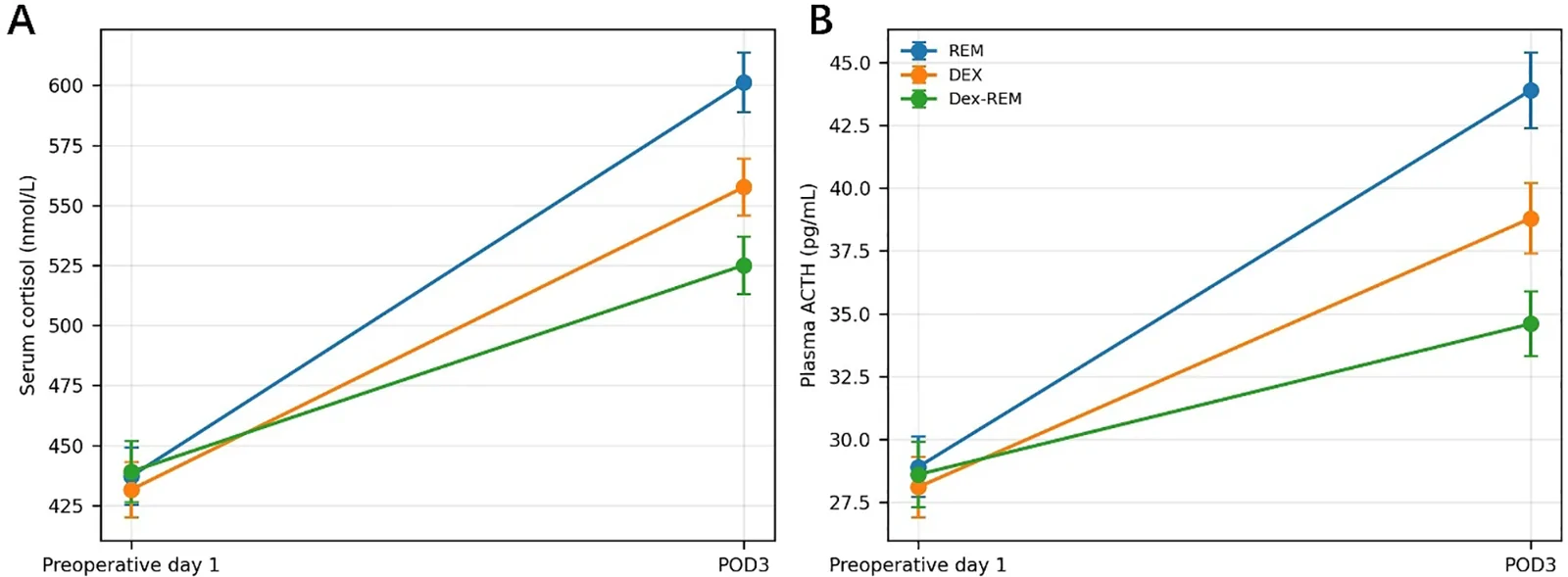
Endocrine biomarker changes at two time points. Mean serum cortisol and plasma ACTH at preoperative day 1 and POD3 by exposure group. Error bars show 95% confidence intervals around group means.

**Table 3:** Adjusted endocrine outcomes.

Outcome	Unadjusted group means	Adjusted contrast	Effect estimate (95% CI)	p-value	Multiplicity note
Primary: cortisol change, nmol/L	REM +164.0; DEX + 126.0; Dex-REM +86.0	Dex-REM vs REM	-78.4 (-103.2 to -53.6)	<0.001	Primary; hierarchical
Primary: cortisol change, nmol/L	REM +164.0; DEX + 126.0; Dex-REM +86.0	Dex-REM vs DEX	-39.6 (-64.1 to -15.1)	0.002	Tested after primary contrast
Cortisol change, nmol/L	REM +164.0; DEX + 126.0; Dex-REM +86.0	DEX vs REM	-38.8 (-63.2 to -14.3)	0.002	Descriptive contrast
ACTH change, pg/mL	REM +15.0; DEX + 10.7; Dex-REM +6.0	Dex-REM vs REM	-8.5 (-10.8 to -6.2)	<0.001	FDR q<0.001
HPA-axis stress burden, SD	REM +0.47; DEX + 0.04; Dex-REM -0.46	Dex-REM vs REM	-0.92 (-1.17 to -0.67)	<0.001	FDR q<0.001
Epinephrine change, pg/mL	REM +58.2; DEX + 54.3; Dex-REM +52.1	Dex-REM vs REM	-6.1 (-15.5 to 3.3)	0.204	FDR q=0.204; exploratory
Norepinephrine change, pg/mL	REM +180.5; DEX + 176.4; Dex-REM +160.1	Dex-REM vs REM	-20.6 (-42.8 to 1.6)	0.069	FDR q=0.083

Effects are adjusted mean differences from linear regression models adjusted for the corresponding baseline biomarker and the prespecified covariate set including age, sex, ASA class, hypertension, clinical tumor stage, baseline hemoglobin, neoadjuvant therapy, and planned surgical approach unless otherwise specified. The primary cortisol endpoint used hierarchical testing. Secondary endocrine outcomes in the FDR family were ACTH, HPA-axis stress burden, epinephrine, and norepinephrine. The HPA-axis stress burden score was exploratory and was calculated as [z(delta cortisol) + z(delta ACTH)]/2 using balanced common-support cohort change-score means and SDs; it is a unitless biological summary, not a validated diagnostic marker.

### Clinical secondary outcomes and neurocognitive status

Selected clinical secondary outcomes were generally favorable for Dex-REM but were more heterogeneous than the primary biomarker result and were interpreted as supportive. Compared with REM, Dex-REM was associated with lower 24-h VAS AUC (aMD, -5.8 point-hours; 95% CI, -9.3 to -2.3; FDR q=0.006) and lower CRP change (aMD, -12.1 mg/L; 95% CI, -23.6 to -0.7; FDR q=0.049). IL-6 change was directionally lower but did not clearly survive FDR control (aMD, -10.6 pg/mL; 95% CI, -20.8 to -0.4; FDR q=0.056), and MAP time-weighted deviation was not statistically definitive (aMD, -1.3 mmHg; 95% CI, -2.9 to 0.3; FDR q=0.133) (**Figure 3**). POD7 screening-based cognitive decline occurred in 51 of 300 patients: 22 in REM, 17 in DEX, and 12 in Dex-REM. The adjusted risk ratio for Dex-REM versus REM was 0.52 (95% CI, 0.26 to 1.05; p=0.071).

**Figure 3:**
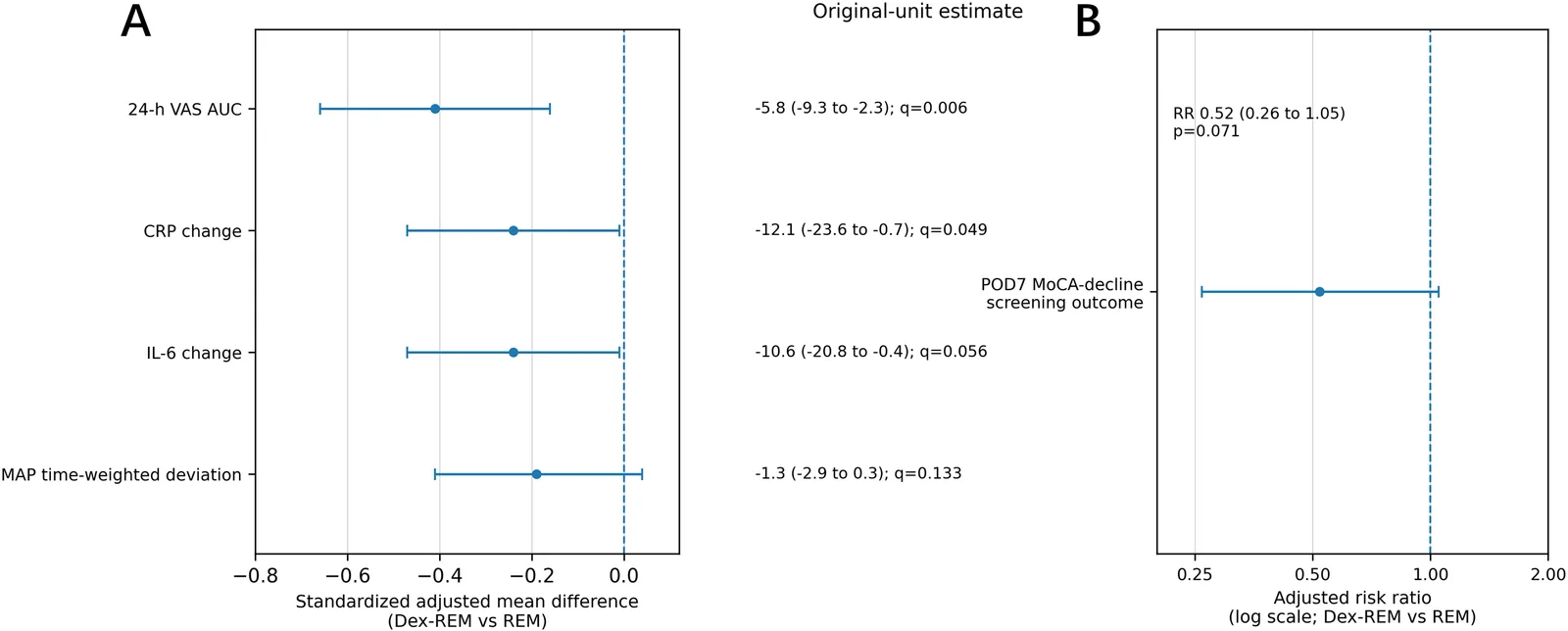
Selected clinical secondary outcomes. **(A)** standardized adjusted mean differences for continuous secondary outcomes, so all continuous effects share a common x-axis; labels show the original-unit adjusted mean difference with 95% CI and FDR q-values. **(B)** the adjusted risk ratio for the POD7 MoCA-decline screening outcome on a log scale with the full CI and a null line at 1.0. Confidence intervals crossing the null indicate statistically imprecise secondary findings.

### Sensitivity analyses and safety

The Dex-REM versus REM cortisol-change contrast remained directionally stable across sensitivity analyses: primary adjusted common-support model, -78.4 nmol/L (95% CI, -103.2 to -53.6); pre-exposure-covariate model, -76.9 nmol/L (95% CI, -101.5 to -52.3); model additionally adjusted for operation duration, blood loss, transfusion, vasopressor use, and regional analgesia, -74.6 nmol/L (95% CI, -99.2 to -49.9); multinomial propensity-score overlap weighting in the full eligible cohort, -70.4 nmol/L (95% CI, -98.5 to -42.3; maximum absolute standardized mean difference improved from 0.30 before weighting to 0.08 after weighting); robust standard-error model, -78.4 nmol/L (95% CI, -104.0 to -52.8); exclusion of 16 patients with recorded systemic steroid exposure before the POD3 blood draw (N = 284), -79.8 nmol/L (95% CI, -106.0 to -53.7); and exclusion of major complications before POD3, -73.2 nmol/L (95% CI, -100.1 to -46.3) (**Table 4**). No clear early descriptive safety signal was observed in Dex-REM, including hypotension (14.0%), bradycardia (10.0%), respiratory depression (5.0%), delayed emergence (7.0%), ICU transfer (4.0%), and serious adverse events (2.0%). These robustness and safety findings support internal consistency while not eliminating residual confounding, selection by outcome availability, or limited power for uncommon events.

**Table 4:** Sensitivity analyses for cortisol change and descriptive safety events.

Analysis or event	Sample or counts	Dex-REM vs REM estimate (95% CI)	p-value/diagnostic
Sensitivity			
Primary common-support covariate-adjusted model	N=300	aMD -78.4 (-103.2 to -53.6)	<0.001
Pre-exposure covariates only	N=300	aMD -76.9 (-101.5 to -52.3)	<0.001
Common-support model plus operation duration, blood loss, transfusion, vasopressor use, and regional analgesia	N=300	aMD -74.6 (-99.2 to -49.9)	<0.001
Multinomial propensity-score overlap weighting in full eligible cohort	N=347	aMD -70.4 (-98.5 to -42.3)	<0.001
Balance diagnostic after overlap weighting	Full eligible cohort	–	Max ASMD before 0.30; after 0.08
Robust HC3 standard errors	N=300	aMD -78.4 (-104.0 to -52.8)	<0.001
Excluding recorded systemic steroid exposure before the POD3 blood draw	N=284 total	aMD -79.8 (-106.0 to -53.7)	<0.001
Excluding major complications before POD3	N=269 total	aMD -73.2 (-100.1 to -46.3)	<0.001
POD3 cortisol as outcome with baseline adjustment	N=300	aMD -78.5 (-103.5 to -53.5)	<0.001
Safety			
Hypotension	REM 15 (15.0); DEX 16 (16.0); Dex-REM 14 (14.0)	–	0.927
Bradycardia	REM 8 (8.0); DEX 11 (11.0); Dex-REM 10 (10.0)	–	0.762
Respiratory depression	REM 8 (8.0); DEX 6 (6.0); Dex-REM 5 (5.0)	–	0.675
Delayed emergence	REM 11 (11.0); DEX 10 (10.0); Dex-REM 7 (7.0)	–	0.599
Postoperative nausea/vomiting	REM 18 (18.0); DEX 14 (14.0); Dex-REM 12 (12.0)	–	0.474
ICU transfer	REM 5 (5.0); DEX 4 (4.0); Dex-REM 4 (4.0)	–	0.923
In-hospital serious adverse event	REM 3 (3.0); DEX 2 (2.0); Dex-REM 2 (2.0)	–	0.864

Sensitivity analyses reported the Dex-REM versus REM adjusted mean difference for cortisol change. ASMD denotes absolute standardized mean difference; the overlap-weighting diagnostic used the full eligible complete-data cohort and reports maximum ASMD before and after weighting. Safety analyses reported early in-hospital safety events as n (%). The steroid-exclusion analysis removed 16 patients with any recorded systemic steroid administration before POD3 sampling; agent, dose, indication, and group-specific counts were not complete standardized fields.

## Discussion

In this complete-case cohort of older patients undergoing radical esophagectomy, the routine Dex-REM anesthetic pathway was associated with a smaller POD3 morning cortisol increase than remifentanil-based anesthesia alone and dexmedetomidine-based anesthesia alone. The primary effect was clinically notable and directionally stable across prespecified sensitivity analyses, and it was accompanied by lower ACTH change and lower exploratory HPA-axis stress burden. Secondary clinical outcomes were not uniformly positive: VAS AUC and CRP favored Dex-REM, whereas IL-6, MAP variability, and POD7 screening-based cognitive decline were directionally favorable but less precise. No clear excess of common early safety events was observed. Because treatment allocation was not randomized, the balanced common-support cohort was selected from naturally unequal exposure strata, and cohort entry required POD3 and POD7 outcome availability, these findings should be interpreted as evidence of association rather than proof that the combined regimen itself caused all observed recovery advantages. The primary endpoint represents POD3 endocrine status after cumulative perioperative care, not a specific surrogate of the intraoperative drug combination or the complete stress-response trajectory.

Surgical trauma activates afferent nociceptive pathways, sympathetic outflow, inflammatory cytokine networks, and HPA-axis signaling, and cortisol is a measurable but nonspecific downstream component of this response (7, 10). Remifentanil may provide strong intraoperative nociceptive suppression, while dexmedetomidine may reduce sympathetic tone, anxiety, and opioid requirements through alpha-2 adrenergic mechanisms (11). Accordingly, the lower POD3 cortisol in the Dex-REM pathway could reflect lower nociceptive burden, direct alpha-2-mediated sympatholysis and upstream HPA-axis modulation, a faster return toward baseline, other postoperative influences, or a combination of these mechanisms. The concordant adjusted ACTH reduction (Dex-REM versus REM, -8.5 pg/mL; 95% CI, -10.8 to -6.2; FDR q<0.001) is compatible with modulation upstream of adrenal cortisol secretion, but two-time-point observational data cannot localize the mechanism or establish a direct drug effect. Lower POD3 cortisol therefore should not be equated with lower surgical stress per se. This biomarker pattern is compatible with prior evidence that dexmedetomidine can attenuate perioperative stress and inflammatory markers (12), but the present design cannot separate pharmacologic synergy from differences in opioid class, opioid dose, anesthesiologist preference, or pathway-related care. The lower CRP and IL-6 changes should not be interpreted as independent causal proof; rather, they support a plausible inflammatory-endocrine recovery pattern that deserves prospective confirmation.

Current nomenclature favors early postoperative cognitive decline or perioperative neurocognitive-disorder terminology rather than the older undifferentiated label of postoperative cognitive dysfunction (18). In the present balanced cohort, POD7 screening-based cognitive decline occurred in 51 patients, and the observed point estimate favored Dex-REM, but the confidence interval crossed 1.0. This direction is compatible with recent literature linking dexmedetomidine or lower inflammatory burden to better early postoperative cognitive recovery (13, 19, 20, 36, 37). However, MoCA change at POD7 is not equivalent to a full neuropsychological diagnosis, and the event count supports only parsimonious modeling. The cognitive result is therefore putative evidence that endocrine and inflammatory attenuation may translate into early functional recovery, not definitive proof of neuroprotection.

The clinical implications are most relevant for perioperative risk stratification and anesthetic pathway design in older esophageal cancer patients. An endocrine-stress-centered framework encourages clinicians and investigators to treat cortisol, ACTH, inflammation, pain burden, and hemodynamic variability as interconnected recovery domains rather than isolated postoperative metrics. If confirmed in a standardized prospective comparison, the Dex-REM pathway could be evaluated in selected older patients in whom nociceptive stress, sympathetic activation, and cognitive vulnerability are major concerns, while maintaining vigilance for bradycardia and hypotension. The absence of a clear observed safety penalty in this cohort is reassuring but not conclusive, because uncommon adverse events require larger multicenter samples. Practical implementation should also consider institutional dosing practices, opioid mixtures, regional analgesia, fluid/vasopressor protocols, and postoperative enhanced-recovery pathways.

The single-center retrospective design is vulnerable to residual confounding by anesthesiologist preference, institutional practice changes, unmeasured frailty, perioperative team behavior, and residual differences in analgesic co-interventions despite partial capture of opioid and binary regional-analgesia exposure. The exposure groups represented clinical anesthetic strategies rather than isolated pharmacologic agents; in particular, the DEX group received more non-remifentanil opioid, and the Dex-REM group cannot be interpreted as a pure synergy model. Important co-interventions, including induction and maintenance agents, anesthetic-depth targets, neuromuscular blockade and reversal, lidocaine, magnesium, technique-level regional analgesia, postoperative dexmedetomidine, and analgesic exposure beyond 24 h, were not available as complete standardized variables; residual confounding by these factors remains. The design also cannot separate direct alpha-2-mediated central HPA-axis modulation from lower nociceptive, inflammatory, or other perioperative stimulation. Although the source population was enumerated and reasons for non-inclusion before the eligible complete-data cohort were reported, the primary analysis still required POD3 cortisol and POD7 MoCA availability. Patients with missing/off-window biomarker sampling, invalid cognitive assessment, clinical instability, prolonged ventilation, ICU escalation, death, early discharge, or transfer may differ systematically from included patients; therefore, selection bias remains possible and may underestimate endocrine or cognitive deterioration in the sickest patients. Because the exposure distribution among the 79 patients who did not enter the eligible pathway cohort was not retrievable, differential outcome availability across anesthetic strategies could bias the group comparison itself. The balanced common-support cohort excluded 47 eligible complete-data cases from the primary analysis; full-cohort overlap-weighted sensitivity analysis partly addressed this tradeoff but cannot fully restore the information lost during selection. Cortisol and ACTH were measured only preoperatively and on POD3. POD3 therefore represents a single early-recovery status point rather than the peak or full HPA-axis trajectory; a smaller rise could reflect attenuation of an earlier response, faster return toward baseline, postoperative influences, or a combination of these. Because the endpoint followed three days of cumulative care, pain, nonuniform postoperative analgesia, inflammation or infection, ICU care, sleep disruption, corticosteroid exposure, and overall recovery may all have contributed. Only total serum cortisol was measured. Postoperative changes in albumin and corticosteroid-binding globulin can alter total cortisol without a proportional change in biologically active free cortisol (38). Although baseline albumin was similar across groups, POD3 albumin, corticosteroid-binding globulin, and free cortisol were unavailable, so binding-protein effects could not be assessed. POD7 cognitive assessment was short-term and based on MoCA change rather than a comprehensive neuropsychological battery with long-term follow-up. Formal serial postoperative sedation scores were unavailable; wake-up time and PACU agitation are proximal recovery descriptors but not substitutes for validated sedation assessment, so an influence of sedation, illness, or concomitant medication on the POD7 MoCA result cannot be excluded. The sample size was adequate for a moderate continuous cortisol endpoint but not for definitive safety comparisons, subgroup effects, or uncommon adverse events. Some sensitivity analyses used total rather than group-specific denominators in the available aggregate table, and secondary findings should remain supportive despite FDR control.

In conclusion, the routine Dex-REM anesthetic pathway was associated with a smaller POD3 morning serum cortisol increase in older radical esophagectomy patients within a retrospective complete-case balanced common-support cohort. This association pertains to the cumulative perioperative pathway and cannot be attributed specifically to dexmedetomidine, remifentanil, their interaction, or the intraoperative strategy independently of postoperative care. Some linked clinical recovery measures were directionally favorable, but secondary findings remained exploratory. The study supports further evaluation of an endocrine-stress-centered perioperative recovery framework. A multicenter prospective cohort or pragmatic randomized trial with serial endocrine sampling, standardized enhanced-recovery protocols, complete follow-up accounting, and longer neurocognitive assessment is warranted.

## Data Availability

The raw data supporting the conclusions of this article will be made available by the authors, without undue reservation.
